# Effects of the pre-existing coronary heart disease on the prognosis of COVID-19 patients: A systematic review and meta-analysis

**DOI:** 10.1371/journal.pone.0292021

**Published:** 2023-10-10

**Authors:** Saikun Wang, Ruiting Zhu, Chengwei Zhang, Yingze Guo, Mengjiao Lv, Changyue Zhang, Ce Bian, Ruixue Jiang, Wei Zhou, Lirong Guo

**Affiliations:** 1 School of Nursing, Jilin University, Changchun, Jilin, China; 2 Department of Anesthesiology, The Second Hospital of Jilin University, Changchun, Jilin, China; 3 The First Hospital of Jilin University, Changchun, Jilin, China; University of Campania Luigi Vanvitelli: Universita degli Studi della Campania Luigi Vanvitelli, ITALY

## Abstract

Although studies have shown severe Coronavirus disease 2019 (COVID-19) outcomes in patients with pre-existing coronary heart disease (CHD), the prognosis of COVID-19 patients with pre-existing CHD remains uncertain primarily due to the limited number of patients in existing studies. This study aimed to investigate the impacts of pre-existing CHD on the prognosis of COVID-19 patients. Five electronic databases were searched for eligible studies. This article focused on cohort and case-control studies involving the prognosis of COVID-19 patients with pre-existing CHD. The meta-analysis was performed using a random effects model. The odds ratios (ORs) and 95% confidence intervals (CIs) were used as valid indicators. The study was registered in PROSPERO with the identifier: CRD42022352853. A total of 81 studies, involving 157,439 COVID-19 patients, were included. The results showed that COVID-19 patients with pre-existing CHD exhibited an elevated risk of mortality (OR = 2.45; 95%CI: [2.04, 2.94], *P* < 0.001), severe/critical COVID-19 (OR = 2.57; 95%CI: [1.98, 3.33], *P* < 0.001), Intensive Care Unit or Coronary Care Unit (ICU/CCU) admission: (OR = 2.75, 95%CI: [1.61, 4.72], *P* = 0.002), and reduced odds of discharge/recovery (OR = 0.43, 95%CI: [0.28, 0.66], P < 0.001) compared to COVID-19 patients without pre-existing CHD. Subgroup analyses indicated that the prognosis of COVID-19 patients with pre-existing CHD was influenced by publication year, follow-up duration, gender, and hypertension. In conclusion, pre-existing CHD significantly increases the risk of poor prognosis in patients with COVID-19, particularly in those male or hypertensive patients.

## Introduction

Coronavirus disease 2019 (COVID-19), caused by severe acute respiratory syndrome coronavirus 2 (SARS-CoV-2) infection [1], has significantly influenced global health, with a staggering 693,664,611 reported cases and 6,908,560 deaths worldwide as of August 18^th^, 23 [2]. Despite most patients having better prognoses, emerging evidence points to vulnerable demographics, studies have shown that including the elderly, obese individuals, and those with pre-existing health conditions like chronic kidney disease, chronic obstructive pulmonary disease, cerebrovascular disease, cancer, and especially cardiovascular disease (CVD) may experience less favorable prognoses [3–7]. Pre-existing CVD, in particular, has been implicated in aggravating pneumonia and elevating mortality [8]. The reported global lethality rate of COVID-19 stands at 1.0% [2], while a previous study of 72,314 cases demonstrated a much higher mortality rate of 10.5% for COVID-19 patients with pre-existing CVD [9]. Among these, coronary heart disease (CHD) emerges as a prominent disease in CVD, frequently associated with severe COVID-19 cases [10].

CHD, characterized by coronary atherosclerosis leading to myocardial hypoxia and necrosis, is typically manifested by plaque formation, narrowing of the coronary artery lumen, and paroxysmal or persistent angina pectoris [11, 12]. Previous studies have shown that patients with CHD are more prone to COVID-19 infection due to reduced cardiac function and diminished immunity [13]. SARS-CoV-2 infection further escalates the probability of acute cardiovascular incidents, contributing to increased severity [14]. Concurrently, CHD patients manifest worsened outcomes from infection including respiratory ailments, and CHD patients with SARS-CoV-2 infection magnify adverse prognosis [15].

Studies have shown that arrhythmias, heart failure, and cardiomyopathy are determinants of poor prognosis in COVID-19 patients [16, 17]. Pre-existing CHD also has been implicated in adverse outcomes in COVID-19 patients [15, 18]. However, the prognostic implications of COVID-19 for patients with pre-existing CHD remain largely undecided and limited by limited patient numbers across these studies. Therefore, the present study was conducted to investigate the effects of pre-existing CHD on the prognosis of patients with COVID-19.

## Materials and methods

### Protocol and search strategy

This systematic review and meta-analysis adhered to the PRISMA (Preferred Reporting Items for Systematic Reviews and Meta-Analyses) guidelines [19]. The study protocol has been registered with PROSPERO under the registration number: CRD42022352853.

PubMed, Scopus, Web of Science, Embase, and Cochrane Library databases were searched to identify relevant studies published up to 10^th^ January 2023. The relevant search strategies employed various combinations of predefined search terms relevant to COVID-19 and CHD, along with Boolean search terms (AND, OR, and NOT). The detailed search strategies are provided in S1 Table. In addition, a manual search of reference lists in the included studies and the relevant reviews was performed to ensure the comprehensiveness of the literature search.

### Inclusion and exclusion criteria

#### Inclusion criteria

(1) Participants were adults with COVID-19 (age ≥ 18 years old). (2) Studies provided information on CHD history (as a comorbidity) in COVID-19 cases. (3) The outcomes of the study included the prognosis of COVID-19 patients (including mortality, severe/critical COVID-19, ICU/CCU admission, or discharge/recovery). (4) Studies were designed as case-control or cohort studies. (5) Studies were published in English.

#### Exclusion criteria

(1) Participants were pregnant. (2) Animal studies. (3) CHD induced by COVID-19 infection. (4) Notes, letters, comments, case reports, and conference abstracts.

Diagnosis of CHD was established based on coronary angiography or other imaging tests indicating coronary artery stenosis or obstruction exceeding 50% [20]. Pre-existing CHD was defined as a reported patient history of CHD upon admission. Severe/critical COVID-19 was defined as patients meeting the following criteria: respiratory rate ≥ 30 breaths/min, resting oxygen saturation ≤ 93%, arterial partial pressure of oxygen (PaO2)/inhaled oxygen concentration (FiO2) ≤ 300 mmHg, or lung imaging depicting > 50% lesion progression within 24 to 48 hours [21].

### Data extraction process

Data from the 81 included articles were extracted independently by two authors. In case of disagreement, a third author was consulted for resolution. The following data were extracted from the included studies: the first author, publication year, participants’ mean/median age, country/region, study design, sample size, gender (the proportion of males), follow-up duration, hypertension history, and primary outcome (mortality, severe/critical COVID-19, ICU/CCU admission, or discharge/recovery). For the primary outcomes, ORs were extracted for subsequent statistical analysis.

### Assessment for quality of studies

Two authors independently assessed the quality of included articles using the Newcastle-Ottawa Scale (NOS) [22], designed for observational studies. Study quality was categorized as low “0–3 points”, moderate “4–6 points”, and high “7–9 points”. Disagreements were resolved with input from a third author.

### Statistical analyses

The extracted data were analyzed by Stata software (version 14.0 SE; Stata Corp LP, College Station, TX, USA) and the Review Manager (Version 5.3. Cochrane Collaboration, Oxford, England). ORs and 95% CIs were calculated using the Mantel-Haenszel formula to assess the effect of pre-existing CHD on the prognosis of patients with COVID-19. OR > 1 indicated pre-existing CHD as a risk factor, whereas OR < 1 indicated it as a protective factor. A 95%CI of the OR included 1 indicated no association. The *I*^*2*^ index was used to assess the heterogeneity of the studies. High heterogeneity (*P* < 0.10 or *I*^*2*^ > 50%) led to the use of the random effect model in the meta-analysis [23]. Subgroup analyses were performed to explore potential sources of heterogeneity and to assess the influence of the region, publication year, study design, NOS score, sample size, age, gender, follow-up duration, and hypertension on the effects of pre-existing CHD on the prognosis of COVID-19 patients. When the number of articles was more than 10, sensitivity analysis was used to assess the impact of individual study on overall significance by excluding each study. Publication bias was assessed using Egger’s linear regression test and the funnel plot analysis [24]. If the publication bias was statistically significant (the funnel plot was visually asymmetric with a *P*-value of Egger’s test < 0.05), the trim and fill method would be performed, and the combined OR would be recalculated. *P*-value < 0.05 indicated statistical significance.

## Results

### Literature search and selection

**Fig 1:**
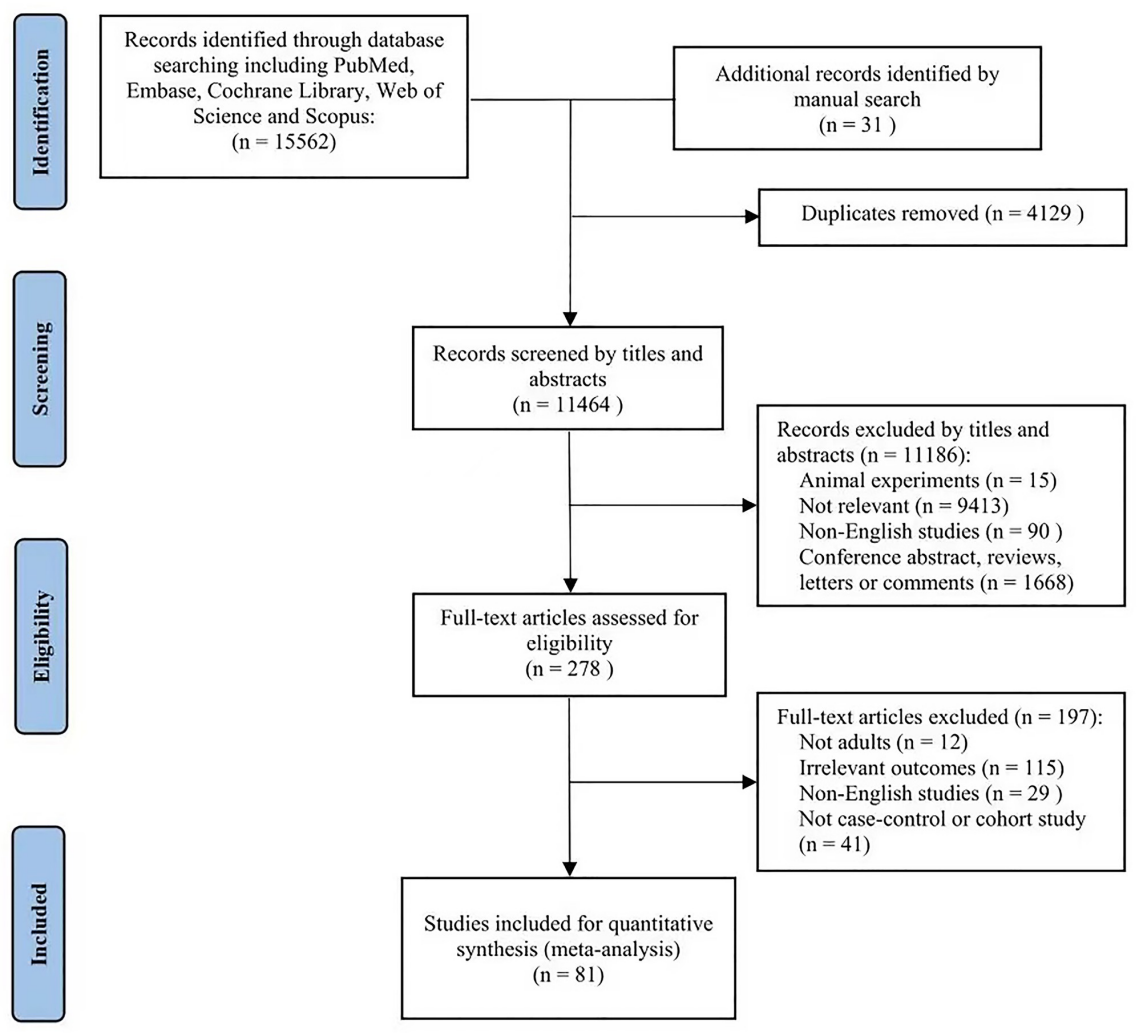
Flow diagram of included studies selection process.

Through a preliminary systematic search, 15,562 literatures were selected from the online database. An additional 31 studies were identified through manual searches. After manually removing duplicates, 11,464 articles remained. Among these, 11,186 articles were excluded after assessing titles and abstracts for relevance to inclusion criteria. Reasons for elimination were as follows: animal experiments; not relevant; non-English publication; conference abstract, reviews, letters, or comments. After full-text evaluation, 278 articles were further excluded due to the following reasons: not being adults (n = 12); irrelevant outcomes (n = 115); non-English studies (n = 29); not case-control or cohort studies (n = 41). Finally, 81 articles [15, 25–104] were included in this systematic review and meta-analysis. The flow of the detailed literature search process was shown in Fig 1.

### Characteristics and quality assessment results of the included studies

Eighty-one studies with 157,439 COVID-19 patients were included in the meta-analysis. These studies, conducted from 2020 to 2022, were distributed across Europe, America, Asia, and Oceania. Eighty-one articles included 51 cohort studies and 30 case-control studies, involving a total of 157,439 COVID-19 patients with sample sizes ranging from 50 to 98,366 individuals. The mean/median age of the participants in the included articles ranged from 40.6 to 71.0 years, with male proportion varying from 35.9% to 79.5%. In addition, the mean/median follow-up duration ranged from 6 to 365 days, and the proportion of hypertension ranged from 6.3% to 82.8%. In the included studies, 44 studies reported mortality as the primary prognosis outcome of COVID-19 patients, 20 studies focused on severe/critical COVID-19, 11 studies focused on ICU/CCU admission, and 6 studies explored discharge/recovery (S2 Table). Quality evaluation results showed that all studies had moderate to high quality with the NOS scores ranging from 6 to 9 points, indicating satisfactory overall quality (S3 Table).

### Pre-existing CHD and mortality

**Fig 2:**
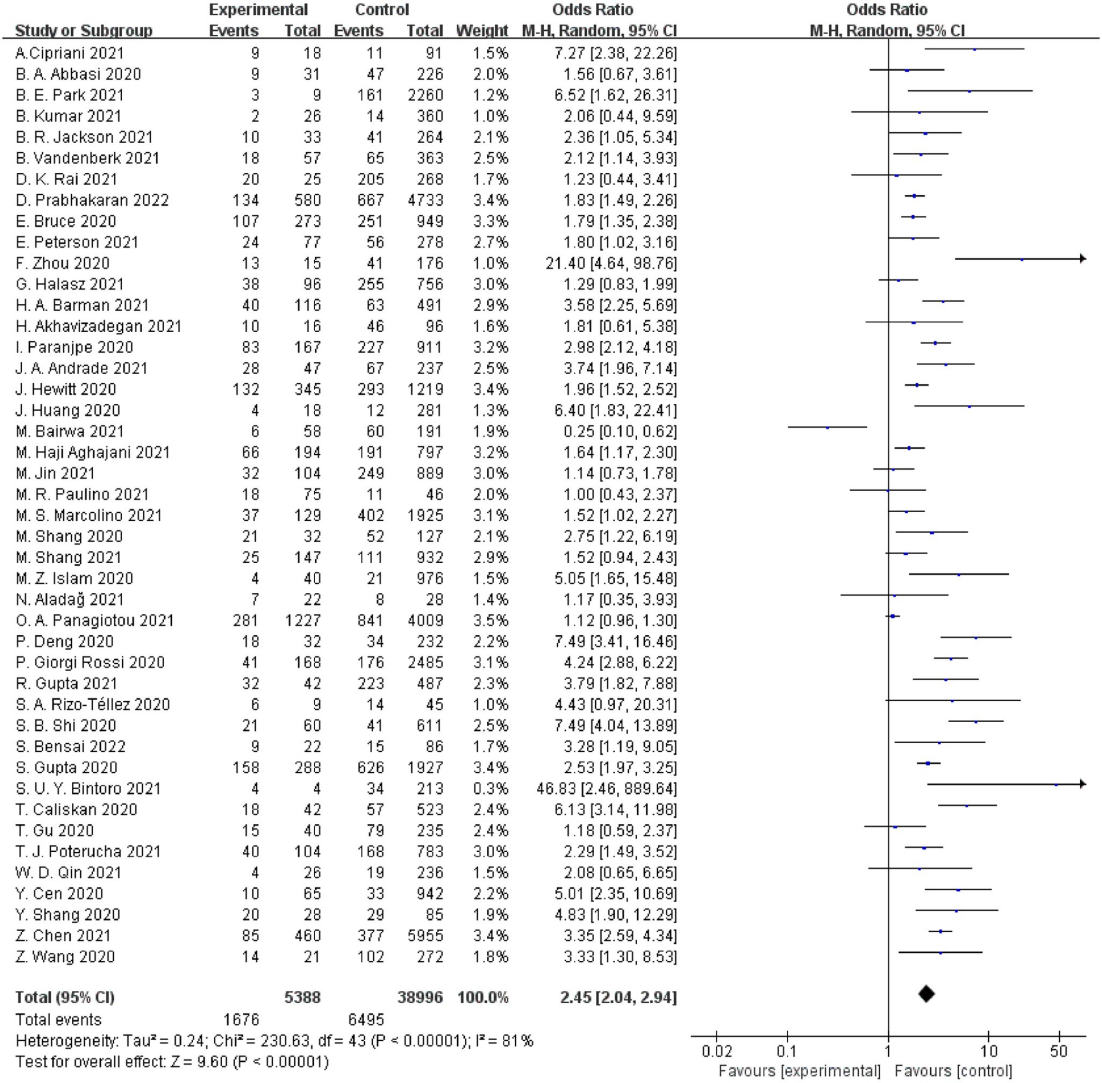
Forest plot indicating the relationship between pre-existing CHD and mortality.

As shown in Fig 2, 44 studies involving 44,384 COVID-19 patients reported mortality as the main outcome. The results of the meta-analysis showed that pre-existing CHD statistically increased the risk of mortality in COVID-19 patients compared to those without pre-existing CHD [the pooled OR = 2.45 (95%CI [2.04, 2.94], P < 0.001), *I*^*2*^ = 81%].

### Pre-existing CHD and severe/critical COVID-19

**Fig 3:**
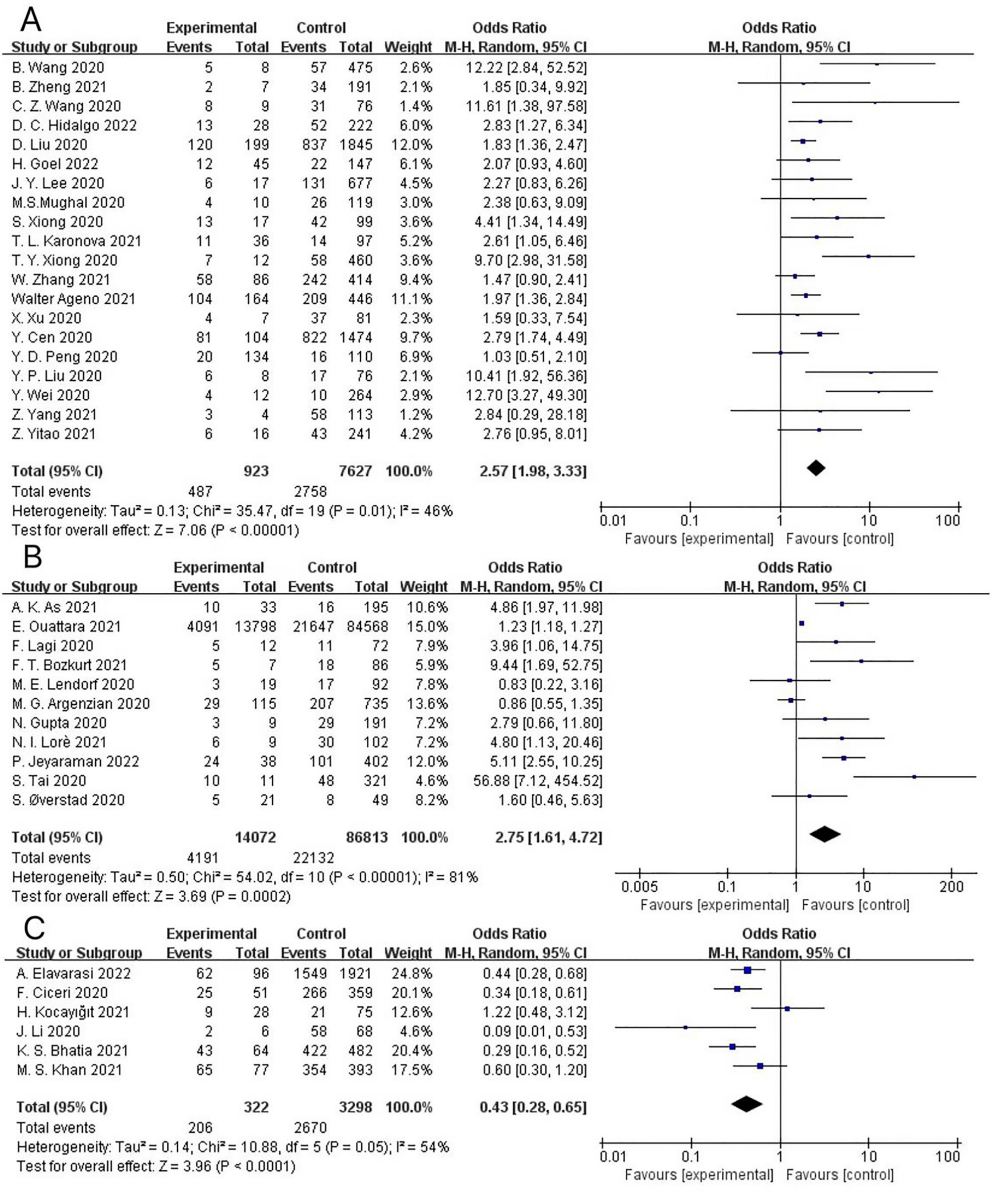
(A) Forest plot indicating the relationship between pre-existing CHD and severe/critical COVID-19. (B) Forest plot indicating the relationship between pre-existing CHD and ICU/CCU admission. (C) Forest plot indicating the relationship between pre-existing CHD and discharge/recovery.

As shown in Fig 3A, 20 studies with 8550 COVID-19 patients reported severe/critical COVID-19 as the primary outcome for participants. The results of the meta-analysis showed that pre-existing CHD notably increased the risk of progressing to severe/critical COVID-19 in COVID-19 patients compared to those without pre-existing CHD [the pooled OR = 2.57 (95%CI [1.98, 3.33], P < 0.001), *I*^*2*^ = 46%].

### Pre-existing CHD and ICU/CCU admission

The primary outcome in 11 studies including 100,885 COVID-19 patients was ICU/CCU admission. The results of the meta-analysis showed that pre-existing CHD substantially increased risks of ICU/CCU admission in COVID-19 patients compared to those without pre-existing CHD [the pooled OR = 2.75 (95%CI [1.61, 4.72], P = 0.002), *I*^*2*^ = 81%] (Fig 3B).

### Pre-existing CHD and discharge/recovery

Six studies involving 3620 COVID-19 patients reported discharge/recovery as the primary outcome. The results of the meta-analysis showed that pre-existing CHD statistically decreased the odds of discharge/recovery in COVID-19 patients compared to COVID-19 patients without pre-existing CHD [the pooled OR = 0.43 (95%CI [0.28, 0.66], P< 0.001), *I*^*2*^ = 54%] (Fig 3C).

### Subgroup analysis

Subgroup analyses were performed to deeper evaluate the results (Table 1).

**Table 1:** Results of subgroup analysis of included studies in the meta-analysis.

Groups	n	mortality			n	severe/critical COVID-19			n	ICU/CCU admission			n	discharge/recovery		
		OR(95%CI)	*I*^*2*^ (%)	*P*-value		OR(95%CI)	*I*^*2*^ (%)	*P*-value		OR(95%CI)	*I*^*2*^ (%)	*P*-value		OR(95%CI)	*I*^*2*^(%)	*P*-value
**Region**																
Europe	10	2.64(1.90,3.67)	76.9	0.000	2	2.05(1.45,2.88)	0.0	0.571	7	3.84(1.54,9.57)	82.4	0.000	1	0.34(0.19,0.61)	0.0	0.000
America	13	2.06(1.58,2.68)	81.6	0.000	3	2.41(1.43,4.07)	0.0	0.862	1	0.86(0.55,1.35)	0.0	0.000	1	0.29(0.16,0.52)	0.0	0.000
Asia	21	2.79(1.94,4.00)	80.6	0.000	15	2.94(2.01,4.29)	59.5	0.002	3	3.40(1.68,6.86)	25.2	0.263	3	0.46(0.16,1.30)	72.8	0.025
Oceania	-	-	-	-	-	-	-	-	-	-	-	-	1	0.30(0.30,1.20)	54.0	0.054
**Publication year**																
2020	17	3.48(2.65,4.57)	75.3	0.000	11	3.71(2.13,6.46)	67.5	0.001	6	2.38(0.90,6.27)	75.6	0.001	2	0.23(0.07,0.76)	48.2	0.165
2021	24	1.96(1.51,2.54)	80.8	0.000	7	2.08(1.65,2.62)	0.0	0.666	4	3.46(1.15,10.38)	83.1	0.000	3	0.56(0.25,1.23)	71.4	0.030
2022	3	1.81(1.50,2.19)	0.0	0.394	2	2.57(1.98,3.34)	0.0	0.586	1	5.11(2.55,10.25)	0.0	0.000	1	0.44(0.28,0.68)	54.0	0.054
**Study design**																
Case-control	16	2.70(2.01,3.61)	72.1	0.000	10	2.37(1.57,3.57)	47.7	0.046	3	10.88(2.46,48.14)	57.4	0.095	1	0.09(0.01,0.54)	0.0	0.000
Cohort study	28	2.34(1.84,2.97)	84.5	0.000	10	2.85(1.97,4.14)	50.8	0.032	8	2.00(1.20,3.33)	78.5	0.000	5	0.45(0.31,0.67)	49.6	0.094
**NOS score**																
<7	18	3.06(2.43,3.85)	69.9	0.000	10	2.37(1.66,3.38)	42.5	0.074	5	5.29(2.03,13.78)	68.4	0.013	3	0.42(0.29,0.59)	20.0	0.287
≥7	26	2.03(1.63,2.54)	76.6	0.000	10	2.94(1.92,4.51)	54.3	0.020	6	1.43(0.96,2.14)	51.3	0.068	3	0.43(0.12,1.31)	76.1	0.015
**Sample size**																
<200	9	3.14(1.79,5.48)	58.7	0.013	9	2.84(1.86,4.33)	0.0	0.642	5	5.28(1.55,18.02)	68.3	0.013	2	0.37(0.03,4.85)	84.4	0.011
≥200	35	2.36(1.94,2.87)	83.6	0.000	11	2.51(1.79,3.50)	64.2	0.002	6	2.02(1.15,3.55)	82.7	0.000	4	0.40(0.30,0.52)	0.0	0.412
**Follow-up time**																
<30	14	2.43(1.94,3.03)	74.4	0.000	10	3.07(1.92,4,92)	0.0	0.843	8	2.52(1.21,5.25)	74.4	0.000	3	0.45(0.22,0.95)	70.9	0.032
≥30	20	2.51(1.80,3.49)	84.3	0.000	8	2.37(1.59,3.53)	55.9	0.016	3	5.13(0.98,26.83)	90.9	0.000	3	0.42(0.23,0.77)	42.2	0.150
Not-reported	10	2.37(1.39,4.03)	78.6	0.000	2	2.45(1.25,4.82)	52.5	0.039	-	-	-	-	-	-	-	-
**Age**																
≤60	13	2.62(1.72,3.99)	83.2	0.000	11	4.41(2.48,7.85)	59.0	0.007	6	5.38(2.77,10.45)	44.5	0.109	1	0.44(0.28,0.68)	0.0	0.000
>60	20	2.65(2.04,3.43)	72.0	0.000	3	1.79(0.88,3.64)	46.0	0.157	4	1.16(0.81,1.67)	47.8	0.125	2	0.28(0.04,1.76)	73.4	0.052
Not-reported	11	2.10(1.51,2.91)	85.4	0.000	6	2.03(1.67,2.47)	0.0	0.793	1	2.79(1.61,4.72)	0.0	0.000	3	0.45(0.22,0.95)	70.9	0.032
**Gender (Male%)**																
≤60	15	1.90(1.30,2.78)	77.1	0.000	3	2.13(1.51,2.99)	52.7	0.006	4	2.10(0.96,4.59)	71.1	0.016	3	0.33(0.15,0.72)	58.9	0.088
>60	27	2.73(2.18,3.40)	83.5	0.000	17	2.69(1.96,3.69)	0.0	0.438	7	3.78(1.39,10.30)	84.0	0.000	2	0.40(0.28,0.57)	0.0	0.482
Not-reported	2	3.27(0.97,11.98)	87.0	0.006	-	-	-	-	-	-	-	-	1	1.22(0.48,3.12)	0.0	0.000
**Hypertension (%)**																
≤30	11	3.76(2.06,6.84)	86.2	0.000	10	4.81(2.78,8.30)	30.5	0.165	3	3.04(1.15,8.00)	25.0	0.263	1	0.44	0.0	0.000
>30	32	2.2(1.83,2.64)	77.6	0.000	10	1.97(1.64,2.38)	8.7	0.362	6	2.58(1.08,6.16)	82.2	0.000	5	0.42	63.1	0.029
Not-reported	1	1.8(1.02,3.16)	0.0	0.000	-	-	-	-	2	7.21(0.17,306.91)	92.4	0.000	-	-	-	-

ICU: Intensive Care Unit; CCU, Coronary Care Unit; OR: odds ratio; CI: confidence interval.

Subgroup analyses indicated that pre-existing CHD was a risk factor for prognosis in patients with COVID-19 regardless of the region, publication year, study design, NOS score, sample size, age, gender, follow-up duration, and hypertension, indicating that the results were solid. Notably, the risk of mortality (OR: 1.81, 95%CI: [1.50, 2.19]) and severe/critical COVID-19 (OR: 2.57, 95%CI: [1.98, 3.34]) were lower in the studies published in 2022, compared to 2020 (mortality: OR: 3.48, 95%CI: [2.65, 4.57]; severe/critical COVID-19: OR: 3.71, 95%CI: [2.13, 6.46]).

Meanwhile, the length of follow-up duration affected the prognosis of COVID-19 patients with pre-existing CHD. Longer follow-up was associated with increased risks of mortality and discharge/recovery, while decreased risks of severe/critical COVID-19. When the follow-up duration was ≥30 days, the risk of mortality increased (OR: 2.51, 95%CI: [1.80, 3.49]), the risk of severe/critical COVID-19 decreased (OR: 2.37, 95%CI: [1.59, 3.53]), and the odds of discharge/ recovery decreased (OR: 0.42, 95%CI: [0.23, 0.77]) in COVID-19 patients with pre-existing CHD, compared with follow-up duration < 30 days (mortality: OR: 2.43, 95%CI: [1.94, 3.03]; severe/critical COVID-19: OR: 3.07, 95%CI: [1.92, 4,92]; discharge/recovery: OR: 0.45, 95%CI: [0.22, 0.95]).

Moreover, male COVID-19 patients with pre-existing CHD had a higher risk of poor prognosis than female patients with pre-existing CHD. When the percentage of men > 60%, the ORs and 95%CIs of mortality, severe/critical COVID-19, ICU/CCU admission, and discharge/recovery were 2.73 [2.18, 3.40], 2.69 [1.96, 3.69], 3.78 [1.39, 10.30] and 0.40 [0.28, 0.57], respectively, which were higher than when the percentage of men ≤ 60% (mortality: 1.90 [1.30, 2.78]; severe/critical COVID-19: 2.13 [1.51, 2.99]; ICU/CCU admission: 1.10 [1.96, 4.59]; discharge/recovery: 0.33 [0.15, 0.72]). In addition, hypertensive COVID-19 patients with pre-existing CHD have a slightly lower risk of poor prognosis than non-hypertensive patients with pre-existing CHD. When the proportion of hypertension > 30%, the ORs and 95%CIs of mortality, severe/critical COVID-19, ICU/CCU admission, and discharge/recovery were 2.20 [1.83, 2.64], 1.97 [1.64, 2.38], 2.58 [1.08, 2.16], 0.42 [0.23, 0.76], respectively, which were lower than the proportion of hypertension ≤ 30% (mortality: 3.76 [2.06, 6.84]; severe/critical COVID-19: 4.81 [2.78, 8.30]; ICU/CCU admission: 3.04 [1.15, 8.00]; discharge/recovery: 0.44 [0.28, 0.68]).

### Sensitivity analysis

**Fig 4:**
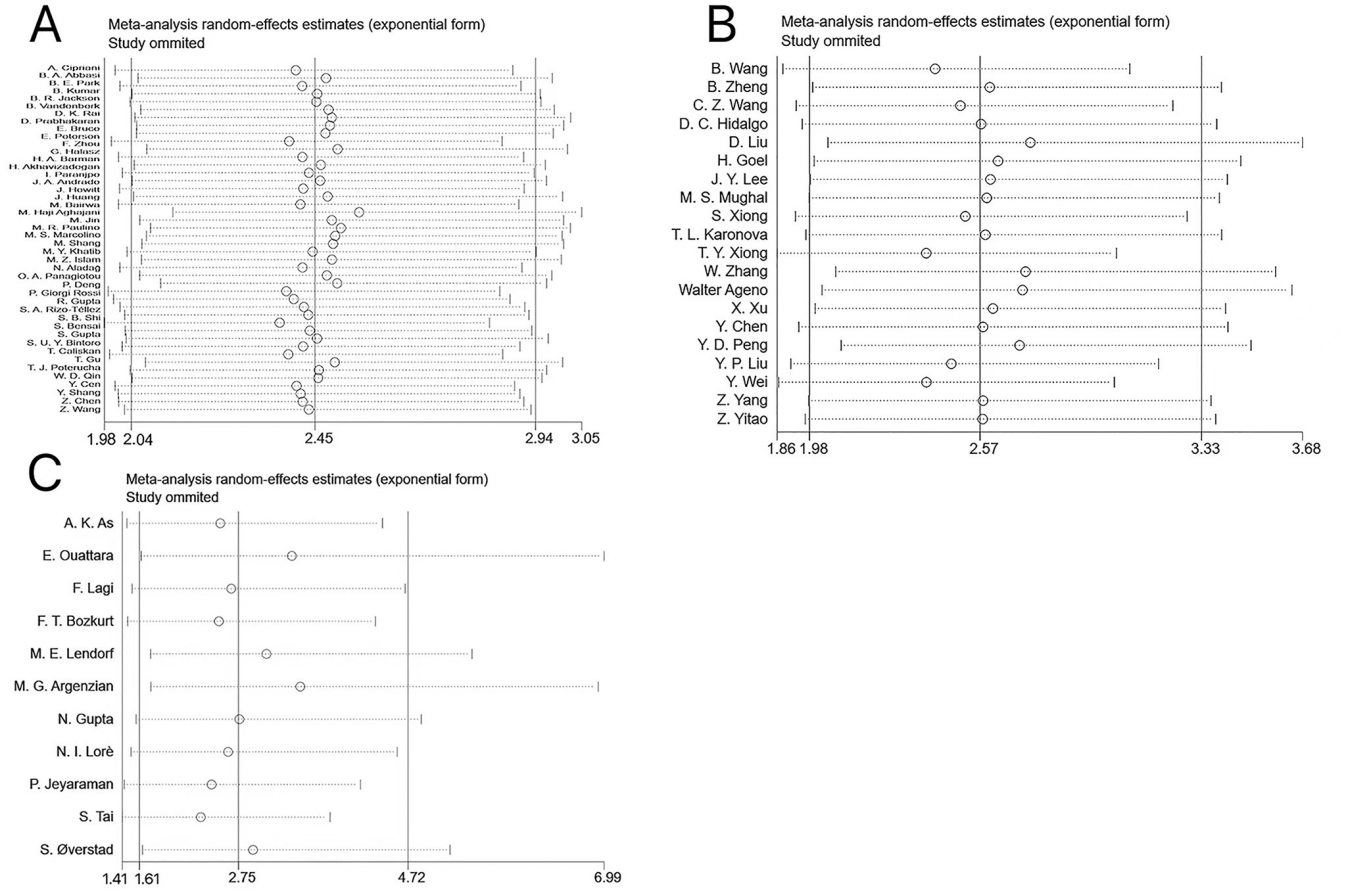
(A) Sensitivity analysis of mortality. (B) Sensitivity analysis of severe/critical COVID-19. (C) Sensitivity analysis of ICU/CCU admission.

The leave-one-out sensitivity analysis was conducted to detect the effect of individual trials on the overall results (Fig 4). The results of sensitivity analysis showed that individual studies included in this study did not alter the overall significance of mortality, severe/critical COVID-19, or ICU/CCU admission, reinforcing the robustness of the association between pre-existing CHD and the poor prognosis of COVID-19 patients. Due to the limited number of studies (only six) with discharge/recovery as the primary outcome, sensitivity analysis was not performed.

### Publication bias and heterogeneity

**Fig 5:**
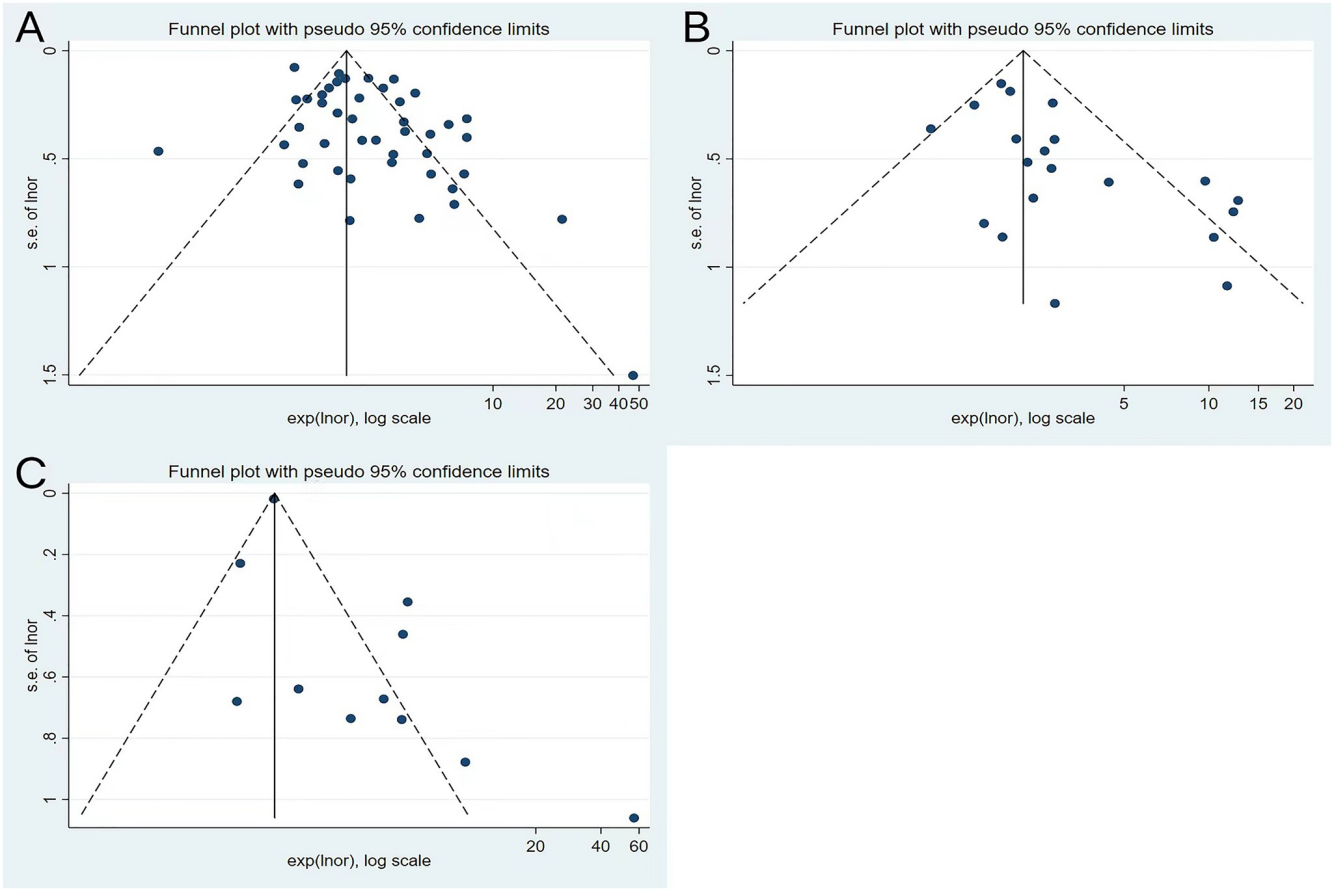
(A) Funnel plot of mortality. (B) Funnel plot of severe/critical COVID-19. (C) Funnel plot of ICU/CCU admission.

Publication bias was analyzed by the funnel plot and Egger’s test. Funnel plots of studies that visually reported mortality, severe/critical COVID-19, and ICU/CCU admissions all showed significant asymmetry, and the *P*-values of the Egger’s test were 0.006, 0.007, and 0.019, respectively, suggesting a potential publication bias (Fig 5).

**Fig 6:**
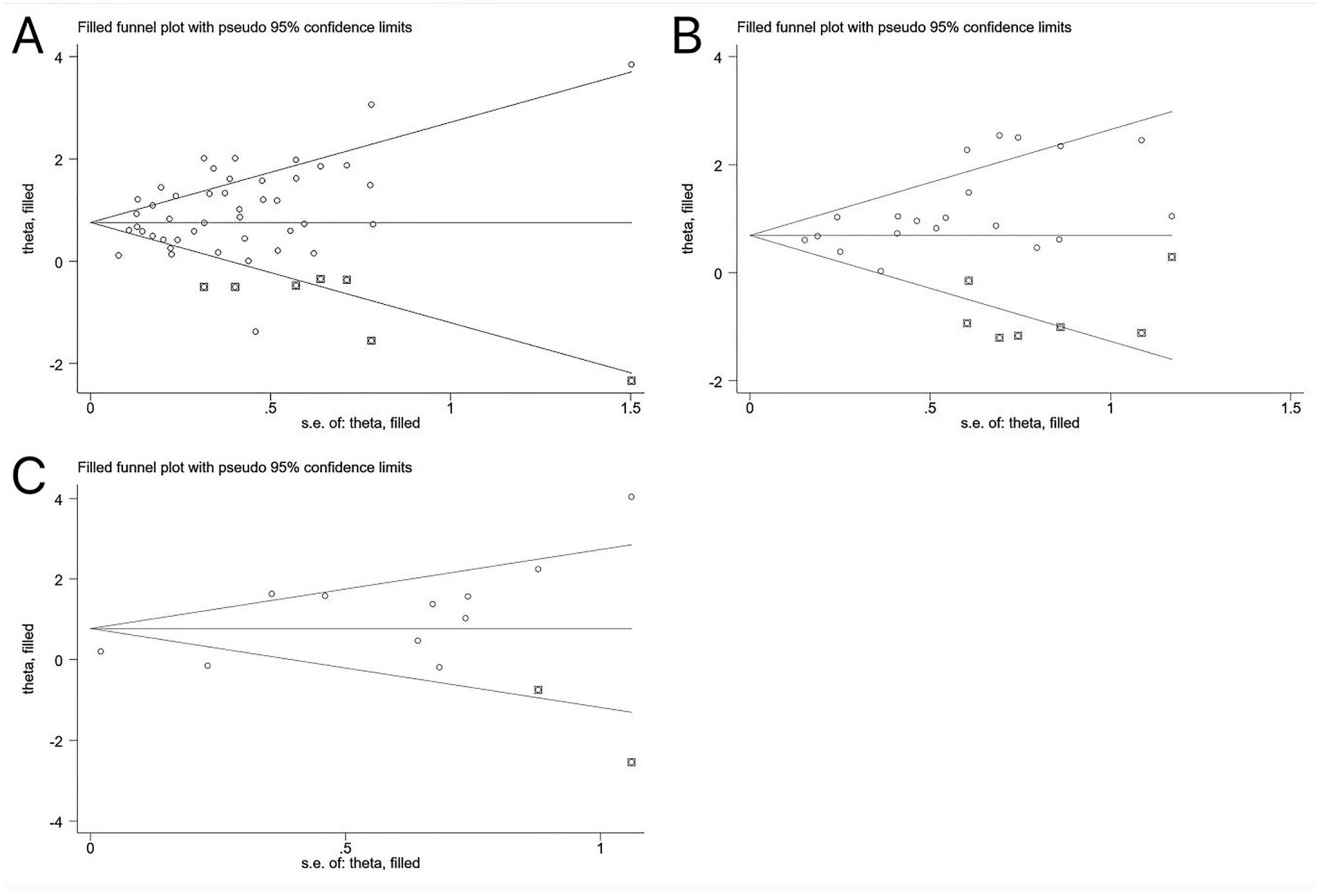
(A) Trim and fill funnel plot of mortality. (B) Trim and fill funnel plot of severe/critical COVID-19. (C) Trim and fill funnel plot of ICU/CCU admission.

Then, the trim and fill method was conducted to detect whether publication bias affected the results (Fig 6). Following the addition of dummy studies, all results remained statistically significant: (mortality: recalculated OR = 2.13, 95%CI [1.78–2.56]; severe/critical COVID-19: recalculated OR = 1.99, 95%CI [1.48–2.68]; ICU/CCU admission: recalculated OR = 2.17, 95%CI [1.28–3.66]), which demonstrated that our results were not affected by publication bias. Given that only six of the included studies had discharge/recovery as the primary outcome, Egger’s test and funnel plot were not performed.

The results of the meta-analysis showed substantial heterogeneity across the various results (mortality: *I*^*2*^ = 81%, severe/critical COVID-19: *I*^*2*^ = 46%, ICU/CCU admission: *I*^*2*^ = 81%, discharge/prognosis: *I*^*2*^ = 54%). Subgroup analysis was performed to detect potential sources of heterogeneity. The results suggested that factors such as region, publication year, study design, sample size, NOS score, gender, follow-up duration, age, and hypertension might be sources of heterogeneity.

## Discussion

The meta-analysis of random-effects models revealed that pre-existing CHD was associated with poor prognosis among COVID-19 patients. COVID-19 patients with pre-existing CHD faced a 1.45-fold higher risk for mortality, a 1.57-fold higher risk for developing severe/critical COVID-19, a 1.75-fold higher risk for ICU/CCU admission, and substantially lower odds of discharge/recovery compared with COVID-19 patients without pre-existing CHD. Importantly, these associations held even after accounting for potential publication bias, highlighting the robustness of the findings.

Our study was motivated by the observed high prevalence of CAD among severe cases of COVID-19 is high [18, 105]. To investigate the specific impacts of pre-existing CHD on the prognosis of COVID-19 patients, we undertook a comparison between COVID-19 patients with and without CHD. Consistent with previous meta-analyses focusing on CAD and its association with severe COVID-19 outcomes [106, 107], the present study confirmed an elevated risk of mortality and critical disease development in the presence of CHD. Remarkably, the unique contribution of our study lies in its exploration of the broader implications of pre-existing CHD on COVID-19 patients. Both studies [106, 107] investigated exploring the prevalence of CAD in deceased and critical COVID-19 patients. However, our study compared COVID-19 patients with pre-existing CHD with COVID-19 patients without CHD to explore the impact of pre-existing CHD on patients’ prognosis. In addition, this study found that pre-existing CHD increased the risk of ICU/CCU admission as highlighted by Liang et al. in their report [108]. Furthermore, this study found that pre-existing CHD reduced the odds of patient discharge/recovery. Meanwhile, on the basis of previous studies, this study thoroughly explored the potential mechanisms of poor prognosis in patients with pre-existing CHD and conducted subgroup analysis to investigate other risk factors for poor prognosis in COVID-19 patients with pre-existing CHD.

A mechanistic understanding of the relationship between pre-existing CHD and COVID-19 outcomes reveals intricate pathways. CHD can contribute to inflammation activation, endothelial dysfunction, and immune signaling irregularities, making patients with pre-existing CHD more susceptible to COVID-19 [13]. Meanwhile, patients with pre-existing CHD infected by COVID-19 are more likely to experience exacerbated myocardial damage, and exacerbating the hypoxia caused by COVID-19. CHD-induced impairment in coronary artery function leads to diminished nutrients and oxygen supply to the contracting heart muscle, culminating in myocardial ischemia [109]. This ischemia triggers sympathetic nervous system activation and cascade involving the circulating renin-angiotensin system, leading to systemic vasoconstriction, irreversible myocardial damage, and reduced cardiac ejection capacity [110]. This cardiac compromise extends to the pulmonary circulation, aggravating the respiratory dysfunction caused by COVID-19, thereby exacerbating cellular hypoxia and ultimately contributing to a poor prognosis for COVID-19 patients [111].

Secondly, endothelial inflammation and fibrosis, frequently seen in CHD lay the groundwork for plaque formation within the arterial wall [12]. The inflammatory state caused by COVID-19 infection can induce plaque rupture in patients with pre-existing CHD through localized inflammation, induction of inflammatory factors, and hemodynamic changes [15]. The plaque rupture exposes potentially thrombogenic elements, provoking acute or subacute thrombosis [112]. These thrombotic events disrupt the equilibrium between myocardial metabolic demand and supply, exacerbating tissue hypoxia, leading to more severe damage, and consequently increasing the risk of poor prognosis in COVID-19 patients [113]. Meanwhile, the thrombosis induces an immune response and increases the production of inflammatory mediators, intensifying the infamous "cytokine storm" [114]. Thus, systemic inflammation and distant organ damage are aggravated, leading to a poor prognosis.

In addition, subgroup analyses showed a reduction in mortality and severe/critical COVID-19 risk associated with studies published in 2022 compared to 2020. This change may be associated with the advancements in COVID-19 vaccines, treatment modalities, and epidemic control strategies [115]. The altered virulence of SARS-CoV-2 might also play a role, rendering them more transmissible but less severe [116]. In addition, longer follow-up durations were associated with increased mortality risk but decreased severe/critical COVID-19 risk and discharge/recovery odds. Compared with follow-up duration < 30 days, when follow-up duration ≥ 30 days, the risk of mortality increased, the risk of severe/critical COVID-19 decreased, and the odds of discharge/recovery decreased. This may be due to incomplete disease progression at shorter follow-up durations and the influence of additional underlying conditions on mortality risk [117]. The reduced risk of severe/critical COVID-19 may be because the majority of studies with follow-up durations ≥ 30 days were published in 2021 and 2022, resulting in a reduced risk of severe COVID-19 due to vaccine rollout and decreased virulence of the virus [115, 116]. Although the virulence of SARS-CoV-2 has decreased, the follow-up duration is insufficient, and the harm and other sequelae of COVID-19 on the organism have not been fully manifested. Therefore, a long follow-up exploration should be conducted in the future to explore the effects of COVID-19 on the organism.

Additionally, male patients with pre-existing CHD faced higher risks, consistent with the higher vulnerability of male COVID-19 patients [118]. This may be related to elevated ACE2 expression in males and a greater prevalence of smoking, a recognized risk factor for adverse COVID-19 outcomes [119, 120]. Conversely, female patients exhibited relatively better prognoses, potentially attributed to their heightened immune responses, which might provide greater protection [121]. Interestingly, hypertensive COVID-19 patients with pre-existing CHD showed a paradoxically lower risk of poor prognosis from a previous study [122]. Subgroup analysis indicated that hypertensive COVID-19 patients with pre-existing CHD have a lower risk of poor prognosis than non-hypertensive patients with pre-existing CHD. This phenomenon might be linked to the use of antihypertensive medicines in hypertensive patients, such as angiotensin-converting enzyme inhibitors (ACEIs) and angiotensin II type 1 receptor blockers (ARBs) medications. ACEIs and ARBs have cardioprotective effects and have been shown to significantly reduce mortality in patients with CHD [123]. Meanwhile, studies have suggested that angiotensin II was elevated in COVID-19 patients compared to healthy individuals [124]. Angiotensin II positively regulates the expression of inflammatory cytokines Excessive levels of inflammatory cytokines are detrimental to the prognosis of COVID-19 patients [125]. ACEI/ARB inhibits angiotensin II expression and improves the prognosis of COVID-19 patients with hypertension. Further studies are needed to investigate the effect of hypertension on the prognosis of COVID-19 patients in the future.

In addition, the potential benefits of statin therapy for patients with pre-existing CHD were highlighted. Statins, renowned for reducing cholesterol levels and atherosclerotic plaque formation, showed potential ability to improve COVID-19 prognosis [126]. Statins have been reported to improve the poor prognosis of COVID-19 due to their beneficial effects in patients with pneumonia and other infectious diseases [127, 128]. Pre-admission statin therapy in COVID-19 patients with pre-existing CHD may improve patient prognosis. This may be due to the anti-inflammatory and immunomodulatory properties of statins. These properties are implicated in the reduction of pro-inflammatory cytokines such as glucagon-1b (IL), IL-6, and tumor necrosis factor-alpha (TNF-α), thereby ameliorating systemic inflammatory status [129]. In addition, statins are associated with reductions in blood lipid levels, preservation of endothelial function, prevention of myocardial damage, and amelioration of hypoxia. These multi-benefits contribute to improving patient prognosis [130]. Prior medication therapy history could play a crucial role in shaping COVID-19 outcomes and need further investigation.

While the present meta-analysis offers several obvious strengths, including its large sample size, robust analytical techniques, and multiple subgroup analyses, some potential limitations also exist. Firstly, heterogeneity was observed but mitigated through sensitivity and subgroup analyses, thus better confirming the validity of this study. Secondly, the lack of comprehensive data on medication therapy (ACEIs, ARBs, statins, and other medications), and vaccination status posed constraints on further analysis in these aspects. Further analysis is needed in the future to explore the effects of medications and vaccines on the prognosis of COVID-19 patients. Thirdly, this meta-analysis only included studies published in English, which may not be comprehensive. Fourthly, the influence of various comorbidities on COVID-19 prognosis could not be comprehensively explored due to limited reporting. Therefore, the effect of different comorbidities on the prognosis of COVID-19 patients with CHD should be further studied in the future. In addition, this study included patients who were diagnosed with CHD prior to admission, but there might have been missed diagnoses. Despite these limitations, the insights derived from this substantial and diverse dataset remain valuable for clinical practice and resource allocation during pandemics.

## Conclusions

In this meta-analysis, we have rigorously investigated the relationship between pre-existing CHD and the prognoses of COVID-19 patients. The pooled evidence indicates a compelling association between pre-existing CHD and poor outcomes in COVID-19 patients. The results from the present study reveal that individuals with pre-existing CHD, especially males or those with hypertension, are faced with a substantially elevated risk of mortality, a heightened susceptibility to severe/critical COVID-19, an increased risk of ICU/CCU admission, and decreased odds of discharge/recovery when compared to their counterparts without CHD.

In conclusion, the present study provides substantial insights that emphasize the critical importance of considering pre-existing CHD as an important factor affecting COVID-19 prognosis. Our study also provides healthcare professionals with valuable knowledge for risk assessment and resource allocation by unraveling the intricate mechanisms and risk associations.

## Supporting information

S1 Table The search strategy used in PubMed/ Scopus/ Web of Science / Cochran library/ Embase online database. (DOCX)

S2 Table Main information extracted from included studies. (DOCX)

S3 Table Newcastle-Ottawa Scale quality assessment of the studies. (DOCX)

S1 Checklist PRISMA checklist. (DOCX)
