# Supplementary material for: Effects of the pre-existing coronary heart disease on the prognosis of COVID-19 patients: A systematic review and meta-analysis
Source: PLoS One. 2023 Oct 10;18(10):e0292021. doi: 10.1371/journal.pone.0292021 (PMC10564240; doi:10.1371/journal.pone.0292021)
Supplement: S1 Table — (DOCX) [file pone.0292021.s001.docx]

**S1 Table. The search strategy used in PubMed/ Scopus/ Web of Science / Cochran library/ Embase online database.**

| **Database name** | **Search strategies: keywords and how these were combined in the search** | **Laster update** | **Number of studies identified** |
| --- | --- | --- | --- |
| PubMed | ((((((((((((((((("Coronary Disease"[Mesh]) OR (coronary disease[Title/Abstract])) OR (coronary diseases[Title/Abstract])) OR (disease, coronary[Title/Abstract])) OR (diseases, coronary[Title/Abstract])) OR (coronary heart disease[Title/Abstract])) OR (CHD[Title/Abstract])) OR (disease,coronary heart[Title/Abstract])) OR (diseases, coronary heart[Title/Abstract])) OR (heart disease, coronary[Title/Abstract])) OR (heart diseases,coronary[Title/Abstract])) OR (coronary artery disease[Title/Abstract])) OR (CAD[Title/Abstract])) OR (artery disease, coronary[Title/Abstract])) OR (artery diseases, coronary[Title/Abstract])) OR (disease, coronary artery[Title/Abstract])) OR (diseases, coronary artery[Title/Abstract])) AND (((((((((((((((((((((((((((((((("COVID-19"[Mesh]) OR (covid 19[Title/Abstract])) OR (COVID-19[Title/Abstract])) (sars-cov-2[Title/Abstract])) OR (sars cov 2 infection[Title/Abstract])) OR (2019 novel coronavirus disease[Title/Abstract])) OR (2019 novel coronavirus infection[Title/Abstract])) OR (2019-ncov disease[Title/Abstract])) OR (2019 ncov disease[Title/Abstract])) OR (2019-ncov diseases[Title/Abstract])) OR (disease, 2019-ncov[Title/Abstract])) OR (covid-19 virus infection[Title/Abstract])) OR (covid-19 virus infections[Title/Abstract])) OR (infection, covid-19 virus[Title/Abstract])) OR (virus infection, covid-19[Title/Abstract])) OR (coronavirus disease 2019[Title/Abstract])) OR (disease 2019, coronavirus[Title/Abstract])) OR (coronavirus disease-19[Title/Abstract])) OR (coronavirus disease 19[Title/Abstract])) OR (severe acute respiratory syndrome coronavirus 2 infection[Title/Abstract])) OR (sars coronavirus 2 infection[Title/Abstract])) OR (covid-19 virus disease[Title/Abstract])) OR (covid-19 virus diseases[Title/Abstract])) OR (disease, covid-19 virus[Title/Abstract])) OR (virus disease, covid-19[Title/Abstract])) OR (2019-ncov infection[Title/Abstract])) OR (2019 ncov infection[Title/Abstract])) OR (2019-ncov infections[Title/Abstract])) OR (infection, 2019-ncov[Title/Abstract])) OR (covid19[Title/Abstract])) OR (covid-19 pandemic[Title/Abstract])) OR (pandemic, covid-19[Title/Abstract])) OR (covid-19 pandemics[Title/Abstract])) | 10th  January  2023 | 1677 |
| Scopus | ( ( TITLE-ABS-KEY ( "Coronary Disease" ) OR TITLE-ABS-KEY ( "Coronary Diseases" ) OR TITLE-ABS-KEY ( "Disease, Coronary" ) OR TITLE-ABS-KEY ( "Diseases, Coronary" ) OR TITLE-ABS-KEY ( "Coronary Heart Disease" ) OR TITLE-ABS-KEY ( chd ) OR TITLE-ABS-KEY ( "Disease, Coronary Heart" ) OR TITLE-ABS-KEY ( "Diseases, Coronary Heart" ) OR TITLE-ABS-KEY ( "Heart Disease, Coronary" ) OR TITLE-ABS-KEY ( "Heart Diseases, Coronary" ) OR TITLE-ABS-KEY ( "Coronary Artery Disease" ) OR TITLE-ABS-KEY ( cad ) OR TITLE-ABS-KEY ( "Artery Disease, Coronary" ) OR TITLE-ABS-KEY ( "Artery Diseases, Coronary" ) OR TITLE-ABS-KEY ( "Disease, Coronary Artery" ) OR TITLE-ABS-KEY ( "Diseases, Coronary Artery" ) ) ) AND ( ( TITLE-ABS-KEY ( covid-19 ) OR TITLE-ABS-KEY ( "COVID 19" ) OR TITLE-ABS-KEY ( sars-cov-2 ) OR TITLE-ABS-KEY ( "SARS CoV 2 Infection" ) OR TITLE-ABS-KEY ( "2019 Novel Coronavirus Disease" ) OR TITLE-ABS-KEY ( "2019 Novel Coronavirus Infection" ) OR TITLE-ABS-KEY ( "2019-nCoV Disease" ) OR TITLE-ABS-KEY ( "2019 nCoV Disease" ) OR TITLE-ABS-KEY ( "2019-nCoV Diseases" ) OR TITLE-ABS-KEY ( "Disease, 2019-nCoV" ) OR TITLE-ABS-KEY ( "COVID-19 Virus Infection" ) OR TITLE-ABS-KEY ( "COVID-19 Virus Infections" ) OR TITLE-ABS-KEY ( "Infection, COVID-19 Virus" ) OR TITLE-ABS-KEY ( "Virus Infection, COVID-19" ) OR TITLE-ABS-KEY ( "Coronavirus Disease 2019" ) OR TITLE-ABS-KEY ( "Disease 2019, Coronavirus" ) OR TITLE-ABS-KEY ( "Coronavirus Disease-19" ) OR TITLE-ABS-KEY ( "Coronavirus Disease 19" ) OR TITLE-ABS-KEY ( "Severe Acute Respiratory Syndrome Coronavirus 2 Infection" ) OR TITLE-ABS-KEY ( "SARS Coronavirus 2 Infection" ) OR TITLE-ABS-KEY ( " COVID-19 Virus Disease" ) OR TITLE-ABS-KEY ( " COVID-19 Virus Diseases" ) OR TITLE-ABS-KEY ( " Disease, COVID-19 Virus" ) OR TITLE-ABS-KEY ( " Virus Disease, COVID-19" ) OR TITLE-ABS-KEY ( " 2019-nCoV Infection" ) OR TITLE-ABS-KEY ( " 2019 nCoV Infection" ) OR TITLE-ABS-KEY ( "2019-nCoV Infections " ) OR TITLE-ABS-KEY ( " Infection, 2019-nCoV" ) OR TITLE-ABS-KEY ( covid19 ) OR TITLE-ABS-KEY ( " COVID-19 Pandemic " ) OR TITLE-ABS-KEY ( " Pandemic, COVID-19 " ) OR TITLE-ABS-KEY ( " COVID-19 Pandemics " ) ) ) | 10th  January  2023 | 2780 |
| Web of Science | #1 Coronary Disease (Topic) or Coronary Diseases (Topic) or Disease, Coronary (Topic) or Diseases, Coronary (Topic) or Coronary Heart Disease (Topic) or CHD (Topic) or Disease, Coronary Heart (Topic) or Diseases, Coronary Heart (Topic) or Heart Disease, Coronary (Topic) or Heart Diseases, Coronary (Topic) or Coronary Artery Disease (Topic) or CAD (Topic) or Artery Disease, Coronary (Topic) or Artery Diseases, Coronary (Topic) or Disease, Coronary Artery (Topic) or Diseases, Coronary Artery (Topic) 414637  #2 COVID-19 (Topic) or COVID 19 (Topic) or SARS-CoV-2 (Topic) or SARS CoV 2 Infection (Topic) or 2019 Novel Coronavirus Disease (Topic) or 2019 Novel Coronavirus Infection (Topic) or 2019-nCoV Disease (Topic) or 2019 nCoV Disease (Topic) or 2019-nCoV Diseases (Topic) or Disease, 2019-nCoV (Topic) or COVID-19 Virus Infection (Topic) or COVID-19 Virus Infections (Topic) or Infection, COVID-19 Virus (Topic) or Virus Infection, COVID-19 (Topic) or Coronavirus Disease 2019 (Topic) or Disease 2019, Coronavirus (Topic) or Coronavirus Disease-19 (Topic) or Coronavirus Disease 19 (Topic) or Severe Acute Respiratory Syndrome Coronavirus 2 Infection (Topic) or SARS Coronavirus 2 Infection (Topic) or COVID-19 Virus Disease (Topic) or COVID-19 Virus Diseases (Topic) or Disease, COVID-19 Virus (Topic) or Virus Disease, COVID-19 (Topic) or 2019-nCoV Infection (Topic) or 2019 nCoV Infection (Topic) or 2019-nCoV Infections (Topic) or Infection, 2019-nCoV (Topic) or COVID19 (Topic) or COVID-19 Pandemic (Topic) or Pandemic, COVID-19 (Topic) or COVID-19 Pandemics (Topic) 376166  #3 #1 AND #2 2281 | 10th  January  2023 | 2281 |
| Cochrane library | #1 MeSH descriptor: [Coronary Disease] explode all trees OR (Coronary Diseases):ti,ab,kw OR (Disease, Coronary):ti,ab,kw OR (Diseases, Coronary):ti,ab,kw OR (Coronary Heart Disease):ti,ab,kw OR (CHD):ti,ab,kw (Word variations have been searched) OR (Disease, Coronary Heart):ti,ab,kw OR (Diseases, Coronary Heart):ti,ab,kw OR (Heart Disease, Coronary):ti,ab,kw OR (Heart Diseases, Coronary):ti,ab,kw OR (Coronary Artery Disease):ti,ab,kw (Word variations have been searched) OR (CAD):ti,ab,kw OR (Artery Disease, Coronary):ti,ab,kw OR (Artery Diseases, Coronary):ti,ab,kw OR (Disease, Coronary Artery):ti,ab,kw OR (Diseases, Coronary Artery):ti,ab,kw 43519  #2 MeSH descriptor: [COVID-19] explode all trees OR (COVID 19):ti,ab,kw OR (SARS-CoV-2):ti,ab,kw OR (SARS CoV 2 Infection):ti,ab,kw OR (2019 Novel Coronavirus Disease):ti,ab,kw OR (2019 Novel Coronavirus Infection):ti,ab,kw OR ("2019-nCoV Disease"):ti,ab,kw OR (2019 nCoV Disease):ti,ab,kw OR ("2019-nCoV Diseases"):ti,ab,kw OR ("Disease, 2019-nCoV"):ti,ab,kw OR ("COVID-19 Virus Infection"):ti,ab,kw OR ("COVID-19 Virus Infections"):ti,ab,kw OR ("Infection, COVID-19 Virus"):ti,ab,kw OR ("Virus Infection, COVID-19"):ti,ab,kw OR (Coronavirus Disease 2019):ti,ab,kw OR (Disease 2019, Coronavirus):ti,ab,kw OR ("Coronavirus Disease-19"):ti,ab,kw OR ("COVID-19 Virus Disease"):ti,ab,kw OR (SARS Coronavirus 2 Infection):ti,ab,kw OR (Coronavirus Disease 19):ti,ab,kw OR (Severe Acute Respiratory Syndrome Coronavirus 2 Infection):ti,ab,kw OR ("COVID-19 Virus Diseases"):ti,ab,kw OR ("Disease, COVID-19 Virus"):ti,ab,kw OR ("Virus Disease, COVID-19"):ti,ab,kw OR ("2019-nCoV Infection"):ti,ab,kw OR ("2019 nCoV Infection"):ti,ab,kw OR ("2019-nCoV Infections"):ti,ab,kw OR ("Infection, 2019-nCoV"):ti,ab,kw OR (COVID19):ti,ab,kw OR ("COVID-19 Pandemic"):ti,ab,kw OR ("Pandemic, COVID-19"):ti,ab,kw OR ("COVID-19 Pandemics"):ti,ab,kw 14076  #3 #1 AND #2 166 | 10th  January  2023 | 166 |
| Embase | #1 'ischemic heart disease'/exp OR 'coronary disease':ti,ab,kw OR 'coronary diseases':ti,ab,kw OR 'disease, coronary':ti,ab,kw OR 'diseases, coronary':ti,ab,kw OR 'coronary heart disease':ti,ab,kw OR 'chd':ti,ab,kw OR 'disease, coronary heart':ti,ab,kw OR 'diseases, coronary heart':ti,ab,kw OR 'heart disease, coronary':ti,ab,kw OR 'heart diseases, coronary':ti,ab,kw OR 'coronary artery disease':ti,ab,kw OR 'cad':ti,ab,kw OR 'artery disease, coronary':ti,ab,kw OR 'artery diseases, coronary':ti,ab,kw OR 'disease, coronary artery':ti,ab,kw OR 'diseases, coronary artery':ti,ab,kw 942158  #2 'coronavirus disease 2019'/exp OR 'covid-19':ti,ab,kw OR 'covid 19':ti,ab,kw OR 'sars-cov-2':ti,ab,kw OR 'sars cov 2 infection':ti,ab,kw OR '2019 novel coronavirus disease':ti,ab,kw OR '2019 novel coronavirus infection':ti,ab,kw OR '2019-ncov disease':ti,ab,kw OR '2019 ncov disease':ti,ab,kw OR '2019-ncov diseases':ti,ab,kw OR 'disease, 2019-ncov':ti,ab,kw OR 'covid-19 virus infection':ti,ab,kw OR 'covid-19 virus infections':ti,ab,kw OR 'infection, covid-19 virus':ti,ab,kw OR 'virus infection, covid-19':ti,ab,kw OR 'coronavirus disease 2019':ti,ab,kw OR 'disease 2019, coronavirus':ti,ab,kw OR 'coronavirus disease-19':ti,ab,kw OR 'coronavirus disease 19':ti,ab,kw OR 'severe acute respiratory syndrome coronavirus 2 infection':ti,ab,kw OR 'sars coronavirus 2 infection':ti,ab,kw OR 'covid-19 virus disease':ti,ab,kw OR 'covid-19 virus diseases':ti,ab,kw OR 'disease, covid-19 virus':ti,ab,kw OR 'virus disease, covid-19':ti,ab,kw OR '2019-ncov infection':ti,ab,kw OR '2019 ncov infection':ti,ab,kw OR '2019-ncov infections':ti,ab,kw OR 'infection, 2019-ncov':ti,ab,kw OR 'covid19':ti,ab,kw OR 'covid-19 pandemic':ti,ab,kw OR 'pandemic, covid-19':ti,ab,kw OR 'covid-19 pandemics':ti,ab,kw 369628  #1 AND #2 8658 | 10th  January  2023 | 8658 |
