# Supplementary material for: Effects of the pre-existing coronary heart disease on the prognosis of COVID-19 patients: A systematic review and meta-analysis
Source: PLoS One. 2023 Oct 10;18(10):e0292021. doi: 10.1371/journal.pone.0292021 (PMC10564240; doi:10.1371/journal.pone.0292021)
Supplement: S2 Table — (DOCX) [file pone.0292021.s002.docx]

**S2 Table. Main information extracted from included studies.**

| **First author** | **Publication year** | **Country/Region** | **Study design** | **Sample (n);(Number of exposure/control)** | **Age (Mean/Median) (years);(Number of exposure/control)** | **Male (%);(Number of exposure/control)** | **Follow-up time(Mean/Median)（days）** | **Hypertension(%);(Number of exposure/control)** | **Primary outcome** | **NOSa score** |
| --- | --- | --- | --- | --- | --- | --- | --- | --- | --- | --- |
| A.Cipriani | 2021 | Italy/Europe | Case-control | 109(20/89) | 71(86/69) | 67(50/70.8) | - | 62.4(80.0/58.4) | Mortality | 7 |
| A. Elavarasi | 2022 | India/Asia | Cohort study | 2017(1611/406) | 47.4(44.8/57.5) | 65.4(64.9/67.5) | 50 | 22.7(20.4/31.5) | Discharge/Recovery | 6 |
| A. K. As | 2021 | Turkey/Europe | Cohort study | 228(26/202) | 53.6(69.7/51.6) | 40.4(42.3/40.1) | 31 | 30.7(50.0/28.2) | ICU/CCUb admission | 6 |
| B. A. Abbasi | 2020 | America/America | Cohort study | 257（56/201) | 63(75/60) | 53(49/54) | - | 54.9(71.0/50.0) | Mortality | 6 |
| B. E. Park | 2021 | Korea/Asia | Case-control | 2269(164/2015) | 55.5(77.1/53.8) | 35.9(52.4/34.6) | - | 28.8(63.1/26.2) | Mortality | 6 |
| B. Kumar | 2021 | India/Asia | Case-control | 386(16/370) | - | 60.4(68.8/60.0) | 42 | 22.3(43.8/21.4) | Mortality | 7 |
| B. R. Jackson | 2021 | America/America | Cohort study | 297(51/246) | - | 50.2(43.1/51.6) | 45 | 67.7(86.3/63.8) | Mortality | 7 |
| B. Wang | 2020 | China/Asia | Case-control | 483(62/421) | 48.4(48.1/48.5) | 45.1(38.7/46.1) | 31 | 7.0(8.1/6.9) | Severe/Critical | 7 |
| B. Zheng | 2021 | China/Asia | Case-control | 198(36/162) | 49.5(70.0/46.9) | 40.4(36.1/41.4) | 10 | 9.6(22.2/6.8) | Severe/Critical | 7 |
| C. Z. Wang | 2020 | China/Asia | Case-control | 85(39/46) | 59.4(65.1/53.6) | 52.9(69.2/39.1) | 35 | 25.9(41.0/13.0) | Severe/Critical | 6 |
| D. C. Hidalgo | 2022 | America/America | Cohort study | 250(65/185) | 60.9(62.6/60.7) | 54.8(55.4/54.6) | 30 | 53.6(58.5/51.9) | Severe/Critical | 8 |
| D. K. Rai | 2021 | India/Asia | Case-control | 293(225/68) | 557.63(60.01/49.73) | 79.5(79.6/79.4) | 40 | 44.0(49.8/25.0) | Mortality | 6 |
| D. Liu | 2020 | China/Asia | Cohort study | 2044(957/1087) | - | 48.9(54.9/43.7) | 42 | 39.7(47.5/32.9) | severe/critical | 6 |
| D. Prabhakaran | 2022 | America/America | Cohort study | 5313(801/4512) | 57.0(64.9/55.6) | 59.4(64.5/83.6) | 44 | 47.3(56.3/38.8) | Mortality | 7 |
| E. Bruce | 2020 | Britain/Europe | Case-control | 1222(358/864） | - | 56.5(29.9/70.1） | 28 | 50.3(55.9/47.9) | Mortality | 7 |
| E. Ouattara | 2021 | France/Europe | Cohort study | 98366(25765/72601) | 71(66/74) | 53.8(65.5/49.7) | 61 | - | ICU/CCUb admission | 7 |
| E. Peterson | 2021 | America/America | Case-control | 355(80/275) | - | - | - | - | Mortality | 7 |
| F. Ciceri | 2020 | Italy/Europe | Cohort study | 410(291/119) | - | 72.9(71.1/77.3) | 14 | 49.9(45.0/60.5) | Discharge/Recovery | 8 |
| F. Lagi | 2020 | Italy/Europe | Case-control | 84(16/68) | 62(67/62) | 65.5(87.5/60.3) | 19 | 36.9(31.3/38.2) | ICU/CCUb admission | 7 |
| F. T. Bozkurt | 2021 | Turkey/Europe | Case-control | 93(23/70) | 42.5(63/34) | 69.9(69.6/70) | 7 | 22.6(34.5/15.7) | ICU/CCUb admission | 6 |
| F. Zhou | 2020 | China/Asia | Cohort study | 191(54/137) | 56(69/52) | 62(70/59) | 22 | 30.0(48.0/23.0) | Mortality | 8 |
| G. Halasz | 2021 | Italy/Europe | Cohort study | 852(293/559) | 70(78/65) | 70(73/69) | 9 | 59(70/53) | Mortality | 6 |

**Continued S2 Table.**

| H. A. Barman | 2021 | Turkey/Europe | Case-control | 607(103/504） | 62.5(69.3/57.5) | 55.0(59.0/54.0) | 30 | 43.8(58.0/40.0) | Mortality | 6 |
| --- | --- | --- | --- | --- | --- | --- | --- | --- | --- | --- |
| H. Akhavizadegan | 2021 | Iran/Asia | Case-control | 112(56/56) | 67.6(68.0/67.1) | 68.8(69.6/67.9) | - | 30.4(26.8/33.9) | Mortality | 6 |
| H. Goel | 2022 | America/America | Cohort study | 192(34/158) | - | 51.0(50.0/51.3) | 86 | 68.8(82.4/65.8) | Severe/Critical | 7 |
| H. Kocayığıt | 2021 | Turkey/Asia | Cohort study | 103(30/73) | - | - | 7 | 60.2(56.7/61.6) | Discharge/Recovery | 8 |
| I. Paranjpe | 2020 | America/America | Cohort study | 1078(310/768) | - | 58.3(61.6/56.8) | 32 | 34.6(45.2/30.3) | Mortality | 6 |
| B. Vandenberk | 2021 | Belgium/Europe | Cohort study | 420(83/337) | - | 57(60.2/56.7) | 365 | 52.6(66.3/49.3) | Mortality | 8 |
| J. A. Andrade | 2021 | America/America | Cohort study | 284(95/189) | 67(72.3/64.3) | 54.6(63.2/50.3) | 43 | 73(78.3/70.4) | Mortality | 7 |
| J. Hewitt | 2020 | Britain/Europe | Cohort study | 1564(425/1139) | - | 57.7(60.0/56.9) | 28 | 51.6(56.4/49.8) | Mortality | 6 |
| J. Huang | 2020 | China/Asia | Cohort study | 299(16/283) | 53.4(69.2/52.5) | 53.5(68.8/52.7) | - | 24.7(68.8/22.3) | Mortality | 7 |
| J. Li | 2020 | China/Asia | Case-control | 74(60/14) | 66(62/71) | 59.5(55.0/78.6) | 41 | 47.3(41.7/71.4) | Discharge/Recovery | 7 |
| J. Y. Lee | 2020 | Korea/Asia | Case-control | 694(137/557) | - | 30.5(41.6/27.8) | - | 18.9(44.6/22.4) | Severe/Critical | 6 |
| K. S. Bhatia | 2021 | Australia/Oceania | Cohort study | 546(465/81) | 62.9(59.6/81.7) | 51.8(51.6/53.1) | 25 | 49.5(45.6/71.6) | Discharge/Recovery | 6 |
| M. Bairwa | 2021 | India/Asia | Cohort study | 249(66/183) | 45.2(52.4/40.4) | 68.7(63.6/70.5) | - | 23.3(33.3/19.7) | Mortality | 7 |
| M. E. Lendorf | 2020 | Denmark/Europe | Cohort study | 111(20/91) | 68(64/69) | 60.4(85/54.9) | 12 | 34.2(45.0/31.9) | ICU/CCUb admission | 6 |
| M. G. Argenzian | 2020 | America/America | Cohort study | 850(236/614) | 63.4(62/64) | 60.1(66.9/57.5) | 23 | 61.8(66.9/59.8) | ICU/CCUb admission | 8 |
| M. Haji Aghajani | 2021 | Iran/Asia | Cohort study | 991(257/734) | 61.6(70.8/58.5) | 54.9(61.1/52.7) | 6 | 6.3(8.6/5.4) | Mortality |  |
| M. Jin | 2021 | China/Asia | Cohort study | 993(281/712) | 68(69/68) | 66.0(68.0/65.2) | 68 | 40.4(38.8/41.0) | Mortality | 8 |
| M. R. Paulino | 2021 | Brazil/America | Cohort study | 121(29/92) | - | 66.1(70.0/65.2) | 180 | 83.5(93.1/80.4) | Mortality | 7 |
| M. S. Khan | 2021 | America/America | Cohort study | 470(419/51) | - | 47.9(46.3/60.8) | 31 | 62.8(60.1/84.3) | Discharge/Recovery | 6 |
| M. S. Marcolino | 2021 | Brazil/America | Cohort study | 2054(439/1553) | 59(70/56) | 52.6(54.4/51.8) | 12 | 52.9(70.6/48.0) | Mortality | 7 |
| M. S. Mughal | 2020 | America/America | Cohort study | 129(30/99) | 63.0(65.5/60.0) | 62.8(83.3/56.6) | 13 | 43.4(46.7/42.4) | Severe/Critical | 7 |
| M. Shang | 2021 | China/Asia | Case-control | 159(73/86) | 68(75/61) | 57.2(64.4/51.2) | 25 | 39.6(52.1/29.1) | Mortality | 6 |
| M. Y. Khatib | 2022 | Qatar/Asia | Cohort study | 1079(136/943) | 50(61/48) | 94.3(93.4/94.4) | 20 | 42.6(61.8/39.9) | Mortality | 8 |
| M. Z. Islam | 2020 | Bangladesh/Asia | Cohort study | 1016(25/991) | - | 64.1(76.0/63.8) | 28 | 14.3(36.0/13.7) | Mortality | 7 |
| N. Aladağ | 2021 | Turkey/Europe | Case-control | 50(15/35) | 64.8(68/68) | 44(40/62.9) | - | 72(73.3/71.4) | Mortality | 7 |

**Continued S2 Table.**

| N. Gupta | 2020 | India/Asia | Cohort study | 200(32/168) | - | 57962.4/57.1) | 11 | 23(34.4/20.8) | ICU/CCUb admission | 7 |
| --- | --- | --- | --- | --- | --- | --- | --- | --- | --- | --- |
| N. I. Lorè | 2021 | Italy/Europe | Cohort study | 111(36/75) | 57.6(61.8/53.8) | 63.1(83.3/53.3) | 23 | 34.2(36.1/33.3) | ICU/CCUb admission | 8 |
| O. A. Panagiotou | 2021 | America/America | Cohort study | 5236(1122/4114) | - | 39.2(40.2/39.2) | 30 | 78.6(78.0/78.8) | Mortality | 7 |
| P. Deng | 2020 | China/Asia | Cohort study | 264(52/212) | 64.5(74.5/62.5） | 49.2(63.5/45.8） | 35 | 37.9(51.9/34.4) | Mortality | 6 |
| P. Giorgi Rossi | 2020 | Italy/Europe | Cohort study | 2635(217/2418) | - | 50.1(10.8/49.0) | 14 | 16.2(40.1/11.6) | Mortality | 6 |
| P. Jeyaraman | 2022 | India/Asia | Cohort study | 440(125/315) | 47.5(56.6/43.9) | 67(78.4/62.5) | 11.4 | 35(62.4/24.1) | ICU/CCUb admission | 6 |
| R. Gupta | 2021 | America/America | Cohort study | 529(255/274) | 70(73/66) | 54(52/48) | 210 | 79(49/51) | Mortality | 7 |
| S. A. Rizo-Téllez | 2020 | Mexico/America | Cohort study | 54(20/34) | 57.3(62.9/54.1) | 51.9(75.0/38.2) | 60 | 33.3(60.0/17.6) | Mortality | 6 |
| S. B. Shi | 2020 | China/Asia | Case-control | 671(62/609) | 63(74/61) | 48.0(56.5/47.1) | 55 | 29.7(59.7/26.6) | Mortality | 6 |
| S. Bensai | 2022 | Italy/Europe | Cohort study | 108(24/84) | 71(77/69） | 72.2（75.0/71.4) | - | 60.2(75.0/55.9) | Mortality | 6 |
| S. Gupta | 2020 | America/America | Cohort study | 2215(784/1431) | 60.5(66.0/57.4) | 64.8(68.4/62.9) | 28 | 59.7(68.9/54.6) | Mortality | 6 |
| S. Øverstad | 2020 | Norway/Europe | Case-control | 70(13/57) | 59(62/57) | 67(77/65) | 12 | - | ICU/CCUb admission | 6 |
| S. Tai | 2020 | China/Asia | Cohort study | 332（58/274) | 51(52/50.5) | 39.8(46.6/38.3) | 26 | 11.4(27.9/7.7) | ICU/CCUb admission | 8 |
| S. U. Y. Bintoro | 2021 | Indonesia/Asia | Cohort study | 217(38/179) | 52.1(58.4/50.8) | 53.5(60.5/52.0) | 45 | 30.4(34.2/29.6) | Mortality | 7 |
| S. Xiong | 2020 | China/Asia | Case-control | 116(55/61) | 58.5(64.0/56.0） | 69.0(69.1/68.9） | 28 | 38.8(47.3/31.1) | Severe/Critical | 7 |
| T. Caliskan | 2020 | Turkey/Europe | Case-control | 565(75/490) | 48(70/44) | - | 55 | 22.7(53.3/18.0) | Mortality | 6 |
| T. Gu | 2020 | China/Asia | Case-control | 275(94/181) | 66.4(70.7/64.2) | 62.9(59.6/64.6) | 40 | 39.6(44.7/37.0) | Mortality | 8 |
| T. J. Poterucha | 2021 | America/America | Cohort study | 887(208/679) | 64.1(75.2/60.7) | 58(60/57.1) | 30 | 61.0(78.8/55.5) | Mortality | 7 |
| T. L. Karonova | 2021 | Russia/Europe | Case-control | 133(25/108) | 52(57/51) | 57(60/57) | - | 45.9(60.0/42.6) | Severe/Critical | 6 |
| T. Y. Xiong | 2020 | China/Asia | Cohort study | 472(65/472) | 43(51/43) | 53.0(58.5/52.1) | 15 | 15.0(33.9/12.0) | Severe/Critical | 6 |
| W. D. Qin | 2021 | China/Asia | Case-control | 262(23/239) | 63.5(69/63) | 46.9(43.5/47.3) | 28 | 35.5(52.2/33.9) | Mortality | 7 |
| W. Zhang | 2021 | China/Asia | Case-control | 500(300/200) | 40.6(52.7/22.6) | 53.6(62.0/41.0） | 14 | 30.6(33.7/26.0) | Severe/Critical | 7 |
| Walter Ageno | 2021 | Italy/Europe | Cohort study | 610(313/297) | - | 70(60.1/58.3) | 8 | 50.3(54.3/46.1) | Severe/Critical | 8 |
| X. Xu | 2020 | China/Asia | Case-control | 88(41/47) | - | 40.9(36.6/44.7) | 11 | 26.1(31.7/21.3) | Severe/Critical | 6 |
| Y. Cen | 2020 | China/Asia | Cohort study | 1007(43/964) | 61(72/68) | 49.0(72.1/47.9) | 28 | 26.8(46.5/25.9) | Mortality | 7 |

**Continued S2 Table.**

| Y. Chen | 2021 | China/Asia | Case-control | 1578(903/675) | - | 48.8(49.8/47.4) | 51 | 31.2(35.3/25.6) | Severe/Critical | 6 |
| --- | --- | --- | --- | --- | --- | --- | --- | --- | --- | --- |
| Y. D. Peng | 2020 | China/Asia | Case-control | 244(36/208) | 61.0(58.0/61.5) | 46.3(50.0/45.7) | 31 | 82.8(77.8/83.7) | Severe/Critical | 6 |
| Y. P. Liu | 2020 | China/Asia | Cohort study | 84(23/61) | 53(67/51) | 56.0(69.6/50.8) | 7 | 19.0(43.5/9.8) | Severe/Critical | 8 |
| Y. Shang | 2020 | China/Asia | Case-control | 113(49/64) | 66.0(73.0/62.0) | 64.6(73.5/57.8) | 34 | 44.2(53.1/37.5) | Mortality | 7 |
| Y. Wei | 2020 | China/Asia | Cohort study | 276(14/262) | 51.0(65.0/50.0) | 56.2(71.4/55.3) | 18 | 17.0(57.1/14.9) | Severe/Critical | 7 |
| Z. Chen | 2021 | China/Asia | Cohort study | 6415(462/5342) | 59(71/57) | 47.2(63.0/45.7) | 15 | 30.2(50.8/28.0) | Mortality | 6 |
| Z. Wang | 2020 | China/Asia | Case-control | 293(116/177) | 59.2(72.5/50.7) | 47.1(56.0/41.2) | - | 31.4(56.9/14.7) | Mortality | 6 |
| Z. Yang | 2021 | China/Asia | Cohort study | 117(61/56） | 50.0(56.0/38.0） | 51.3(55.7/46.4） | 60 | 27.4(42.6/10.7) | Severe/Critical | 6 |
| Z. Yitao | 2021 | China/Asia | Cohort study | 257(49/208） | 46.0(60.0/43.0） | 54.0(11.0/43.0) | 20 | 20.6(40.8/15.9） | Severe/Critical | 6 |
| a NOS: Newcastle-Ottawa Scale; b ICU: Intensive Care Unit; CCU: Coronary Care Unit. | | | | | | | | | | |
