# Supplementary material for: Effects of the pre-existing coronary heart disease on the prognosis of COVID-19 patients: A systematic review and meta-analysis
Source: PLoS One. 2023 Oct 10;18(10):e0292021. doi: 10.1371/journal.pone.0292021 (PMC10564240; doi:10.1371/journal.pone.0292021)
Supplement: S3 Table — (DOCX) [file pone.0292021.s003.docx]

**S3 Table. Newcastle-Ottawa Scale quality assessment of the studies.**

| **First author** | **Publication year** | **Study design** | **Selection** | **Comparability** | **Outcome** | **Total score** |
| --- | --- | --- | --- | --- | --- | --- |
| A.Cipriani | 2021 | CC | *** | ** | ** | 7 |
| A. Elavarasi | 2022 | CS | *** | * | ** | 6 |
| A. K. As | 2021 | CS | *** | * | ** | 6 |
| B. A. Abbasi | 2020 | CS | *** | * | ** | 6 |
| B. E. Park | 2021 | CC | *** | * | ** | 6 |
| B. Kumar | 2021 | CC | **** | * | ** | 7 |
| B. R. Jackson | 2021 | CS | *** | ** | ** | 7 |
| B. Wang | 2020 | CC | *** | ** | ** | 7 |
| B. Zheng | 2021 | CC | *** | * | *** | 7 |
| C. Z. Wang | 2020 | CC | *** | * | ** | 6 |
| D. C. Hidalgo | 2022 | CS | **** | * | *** | 8 |
| D. K. Rai | 2021 | CC | *** | * | ** | 6 |
| D. Liu | 2020 | CS | *** | * | ** | 6 |
| D. Prabhakaran | 2022 | CS | *** | ** | ** | 7 |
| E. Bruce | 2020 | CC | *** | ** | ** | 7 |
| E. Ouattara | 2021 | CS | **** | * | ** | 7 |
| E. Peterson | 2021 | CC | *** | ** | ** | 7 |
| F. Ciceri | 2020 | CS | **** | * | *** | 8 |
| F. Lagi | 2020 | CC | *** | * | *** | 7 |
| F. T. Bozkurt | 2021 | CC | *** | * | ** | 6 |
| F. Zhou | 2020 | CS | *** | ** | *** | 8 |
| G. Halasz | 2021 | CS | *** | * | ** | 6 |
| H. A. Barman | 2021 | CC | *** | * | ** | 6 |
| H.Akhavizadegan | 2021 | CC | *** | * | ** | 6 |
| H. Goel | 2022 | CS | **** | * | ** | 7 |
| H. Kocayığıt | 2021 | CS | *** | ** | *** | 8 |
| I. Paranjpe | 2020 | CS | *** | * | ** | 6 |
| B. Vandenberk | 2021 | CS | **** | * | *** | 8 |
| J. A. Andrade | 2021 | CS | *** | * | *** | 7 |
| J. Hewitt | 2020 | CS | *** | * | ** | 6 |
| J. Huang | 2020 | CS | *** | * | *** | 7 |
| J. Li | 2020 | CC | *** | * | *** | 7 |
| J. Y. Lee | 2020 | CC | *** | * | ** | 6 |
| K. S. Bhatia | 2021 | CS | *** | * | ** | 6 |
| M. Bairwa | 2021 | CS | *** | * | *** | 7 |
| M. E. Lendorf | 2020 | CS | *** | * | ** | 6 |
| M. G. Argenzian | 2020 | CS | *** | ** | *** | 8 |
| M. Haji Aghajani | 2021 | CS | *** | ** | *** | 8 |
| M. Jin | 2021 | CS | **** | * | *** | 8 |
| M. R. Paulino | 2021 | CS | *** | * | *** | 7 |
| M. S. Khan | 2021 | CS | *** | * | ** | 6 |

**Continued S3 Table.**

| M. S. Marcolino | 2021 | CS | **** | * | ** | 7 |
| --- | --- | --- | --- | --- | --- | --- |
| M. S. Mughal | 2020 | CS | **** | * | ** | 7 |
| M. Shang | 2021 | CC | *** | * | ** | 6 |
| M. Y. Khatib | 2022 | CS | *** | ** | *** | 8 |
| M. Z. Islam | 2020 | CS | **** | * | ** | 7 |
| N. Aladağ | 2021 | CC | *** | * | *** | 7 |
| N. Gupta | 2020 | CS | **** | * | ** | 7 |
| N. I. Lorè | 2021 | CS | *** | ** | *** | 8 |
| O. A. Panagiotou | 2021 | CS | **** | * | ** | 7 |
| P. Deng | 2020 | CS | *** | * | ** | 6 |
| P. Giorgi Rossi | 2020 | CS | *** | * | ** | 6 |
| P. Jeyaraman | 2022 | CS | *** | * | ** | 6 |
| R. Gupta | 2021 | CS | **** | * | ** | 7 |
| S. A. Rizo-Téllez | 2020 | CS | *** | * | ** | 6 |
| S. B. Shi | 2020 | CC | *** | * | ** | 6 |
| S. Bensai | 2022 | CS | *** | * | ** | 6 |
| S. Gupta | 2020 | CS | *** | * | ** | 6 |
| S. Øverstad | 2020 | CC | *** | * | ** | 6 |
| S. Tai | 2020 | CS | **** | * | *** | 8 |
| S. U. Y. Bintoro | 2021 | CS | **** | * | ** | 7 |
| S. Xiong | 2020 | CC | *** | * | *** | 7 |
| T. Caliskan | 2020 | CC | *** | * | ** | 6 |
| T. Gu | 2020 | CC | **** | * | *** | 8 |
| T. J. Poterucha | 2021 | CS | *** | * | *** | 7 |
| T. L. Karonova | 2021 | CC | *** | * | ** | 6 |
| T. Y. Xiong | 2020 | CS | *** | * | ** | 6 |
| W. D. Qin | 2021 | CC | *** | * | *** | 7 |
| W. Zhang | 2021 | CC | *** | ** | ** | 7 |
| Walter Ageno | 2021 | CS | **** | * | *** | 8 |
| X. Xu | 2020 | CC | *** | * | ** | 6 |
| Y. Cen | 2020 | CS | *** | ** | ** | 7 |
| Y. Chen | 2021 | CC | *** | * | ** | 6 |
| Y. D. Peng | 2020 | CC | *** | * | ** | 6 |
| Y. P. Liu | 2020 | CS | *** | ** | *** | 8 |
| Y. Shang | 2020 | CC | *** | * | *** | 7 |
| Y. Wei | 2020 | CS | **** | * | ** | 7 |
| Z. Chen | 2021 | CS | *** | * | ** | 6 |
| Z. Wang | 2020 | CC | *** | * | ** | 6 |
| Z. Yang | 2021 | CS | *** | * | ** | 6 |
| Z. Yitao | 2021 | CS | *** | * | ** | 6 |
| CC: Case-control; CS: Cohort study | | | | | | |
